# COX-2 but Not mPGES-1 Contributes to Renal PGE2 Induction and Diabetic Proteinuria in Mice with Type-1 Diabetes

**DOI:** 10.1371/journal.pone.0093182

**Published:** 2014-07-01

**Authors:** Zhanjun Jia, Ying Sun, Shanshan Liu, Ying Liu, Tianxin Yang

**Affiliations:** 1 Department of Internal Medicine, University of Utah and Veterans Affairs Medical Center, Salt Lake City, Utah, United States of America; 2 Institute of Hypertension, Sun Yat-sen University School of Medicine, Guangzhou, China; Emory University, United States of America

## Abstract

Prostaglandin E2 (PGE2) has been implicated to play a pathogenic role in diabetic nephropathy (DN) but its source remains unlcear. To elucidate whether mPGES-1, the best characterized PGE2 synthase, was involved in the development of DN, we examined the renal phenotype of mPGES-1 KO mice subjected to STZ-induced type-1 diabetes. After STZ treatment, mPGES-1 WT and KO mice presented the similar onset of diabetes as shown by similar elevation of blood glucose. Meanwhile, both genotypes of mice exhibited similar increases of urinary and renal PGE2 production. In parallel with this comparable diabetic status, the kidney injury indices including the urinary albumin excretion, kidney weight and the kidney histology (PAS staining) did not show any difference between the two genotypes. By Western-blotting and quantitative qRT-PCR, mPGES-1, mPGES-2, cPGES and 15-hydroxyprostaglandin dehydrogenase (15-PGDH) remain unaltered following six weeks of diabetes. Finally, a selective COX-2 inhibitor celecoxib (50 mg/kg/day) was applied to the STZ-treated KO mice, which resulted in significant reduction of urinary albumin excretion (KO/STZ: 141.5±38.4 vs. KO/STZ + Celebrex: 48.7±20.8 ug/24 h, p<0.05) and the blockade of renal PGE2 induction (kidney: KO/STZ: 588.7±89.2 vs. KO/STZ + Celebrex: 340.8±58.7 ug/24 h, *p*<0.05; urine: KO/STZ 1667.6±421.4 vs. KO/STZ + Celebrex 813.6±199.9 pg/24 h, *p*<0.05), without affecting the blood glucose levels and urine volume. Taken together, our data suggests that an as yet unidentified prostaglanind E synthase but not mPGES-1 may couple with COX-2 to mediate increased renal PGE2 sythsesis in DN.

## Introduction

PGE2 is an important modulator of renal physiology involving renin release, hemodynamics, and tubular salt and water transport [1]. Numerous studies proved that renal cyclooxygenase (COX)-2 activity and PGE2 production were increased in diabetes mellitus [2]–[8], which contributed to the pathogenesis of DN [2]–[8]. COX-2 inhibition has been shown to be renoprotective, attenuating glomerulosclerosis and albuminuria and glomerular hypertrophy in STZ-diabetic rats [9], [10]. Moreover, the profile of renal PGE2 receptor (EP_1_–EP_ 4_) expression was altered in STZ diabetic mice [11] and EP1-selective antagonist strikingly retarded the progression of nephropathy as shown by significant improvement of mesangial expansion, glomerular hypertrophy and proteinuria [12]. Most recently, a study demonstrated that EP4 agonism exacerbated kidney injury in a mouse model of STZ-induced type-1 diabetes [13]. All these findings indicated that COX-2-PGE2–EP signaling pathway plays an important role in the development of DN.

However, COX-2 inhibitors have been shown to be associated with increased cardiovascular incidence possibly due to the inhibition of prostaglandin I_2_ [14], [15]. Therefore, long-term treatment of COX-2 inhibitors in diabetic patients is not applicable. In this regard, developing new drugs specifically targeting PGE2 synthases is of vital importance for the DN treatment, as well as other diseases. To date, three forms of PGE2 synthases have been cloned and characterized: microsomal prostaglandin E synthase-1 (mPGES-1), microsomal prostaglandin E synthase-2 (mPGES-2) and cytosolic PGES (cPGES). Among them, mPGES-1 is the best-characterized PGES [16], [17] and serves as a promising target for developing the next generation of analgesics. In past decade, numerous convincing studies demonstrated the critical role of mPGES-1 in mediating PGE2 production under baseline or stimulatory conditions [16]–[24]. Recently, evidence showed that deletion of mPGES-2 or cPGES in mice did not reduce PGE2 levels in vivo [25]–[27] **,** which argues against their PGE2 synthetic property but highlights the importance of mPGES-1 as a PGE2 synthase. In present study, mPGES-1 KO mice were employed to define: 1) if mPGES-1 is the enzymatic source of renal PGE2 production in STZ-induced type I diabetes; 2) if mPGES-1 contributes to the glomerular injury of type-1 diabetes.

## Methods

### Animals

mPGES-1 mutant mice were originally generated by Trebino et al.[28]. This mouse colony was propagated at the University of Utah and maintained on a mixed DBA/1lacJxC57/BL6x129/Sv background. In all studies, 3- to 4-mo-old male mice were used. All mice were maintained under a 12∶12-h light-dark cycle (lights on at 6∶00 a.m. and lights off at 6∶00 p.m.). This study was approved by the University of Utah Institutional Animal Care and Use Committee.

### Induction of type I diabetic mouse model by STZ

After 6-hour fasting, mPGES-1 WT and KO mice were administered with STZ at the dose of 120 mg/kg bodyweight by i.p. injection. STZ was dissolved in citrate buffer (pH 4.5) and injected within 10 min of its dissolution. On fourth and sixth week, mice were placed in metabolic cages (Hatteras Instruments) to collect urine. Then 24-hour urine output and water intake were measured. On the next day of urine collection, the six-hour fasting glucose was examined by using a blood glucose meter (Bayer’s Contour). At the end of the experiment, the kidney was harvested for analysis of protein and gene expression and PAS staining. In a separate experiment, mPGES-1 KO mice were treated by STZ with or without celebrex (50 mg/kg/day in diet) for six weeks.

### Immunoblotting

The whole kidney was lysed and protein concentration was determined by Coomassie reagent. Protein (60 µg) from whole kidney lysates were denatured in boiling water for 10 min, separated by SDS-polyacrylamide gel electrophoresis, and transferred onto nitrocellulose membranes. The blots were blocked overnight with 5% nonfat dry milk in Tris-buffered saline (TBS), followed by incubation for 1 h with rabbit anti-mPGES-1 (Cayman Chemicals), anti-mPGES-2 (Cayman Chemicals), anti-cPGES (Cayman Chemicals) or anti-15-PGDH (Cayman Chemicals) at a dilution of 1∶1000. After being washed with TBS, blots were incubated with a goat anti-horseradish peroxidase-conjugated secondary antibody (1∶1000 dilution) and visualized with ECL kits (Amersham, Piscataway, NJ USA).

### qRT-PCR

Total RNA isolation and reverse transcription were performed as previously described [29]. Oligonucleotides were designed using Primer3 software (available at http://frodo.wi.mit.edu/primer3/) and the sequences are: mPGES-1, 5′-AGCA CACTGCTGGTCATCAA-3′ (sense) and 5′-CTCCACATCTGGGTCACTCC-3′ (antisense) (GenBank accession no. BC024960); mPGES-2, 5′-GCTGGGGCTGTACCACAC-3′ (sense) and 5′-GATTCACCTCCACCACCTGA-3′ (antisense) (GenBank accession no. NM 133783); cPGES, 5′-GGTAGAGACCGCCGGAGT-3′ (sense) and 5′-TCGTACCACTTTGCAGAAGCA-3′ (antisense) (GenBank accession no. NM 019766); 15-PGDH, 5′-GTTCGTCCAGTGTGATGTGG-3′ (sense) and 5′-CCTTCACCTCCGTTTTGCTT-3′(antisense) (GenBank accession no. NM 008278); β-actin, 5′-GCTCTGGCTCCTAGCACCAT-3′ (sense) and 5′-GCCACCGATCCACACAGAGT-3′ (antisense) (GenBank accession no. NM_007393). qPCR amplification was performed using the SYBR Green Master Mix (Applied Biosystems, Warrington, UK) and the Prism 7500 Real-Time PCR Detection System (Applied Biosystems, Foster City, CA, USA). Cycling conditions were 95°C for 10 min, followed by 40 repeats of 95°C for 15 s, and 60°C for 1 min.

### Enzyme Immunoassay

Urine samples were centrifuged for 5 minutes at 10,000 rpm. The whole kidney was homogenized in phosphate-buffered saline and then centrifuged for 5 min at 10,000 r.p.m. The supernatant was diluted 1∶50 with enzyme immunoassay buffer. Concentrations of PGE_2_ were determined by enzyme immunoassay according to manufacturer’s instructions (Cayman Chemicals). Urine albumin was determined using a murine microalbuminuria enzyme-linked immunosorbent assay kit (EXOCELL).

### Statistical Analysis

All values are presented as mean ± SE. Statistical analysis was performed using a Student *t* test or ANOVA. Differences were considered to be significant when *P*<0.05.

## Results

### Evaluation of the onset of diabetes in mPGES-1 WT and KO mice

**Figure 1:**
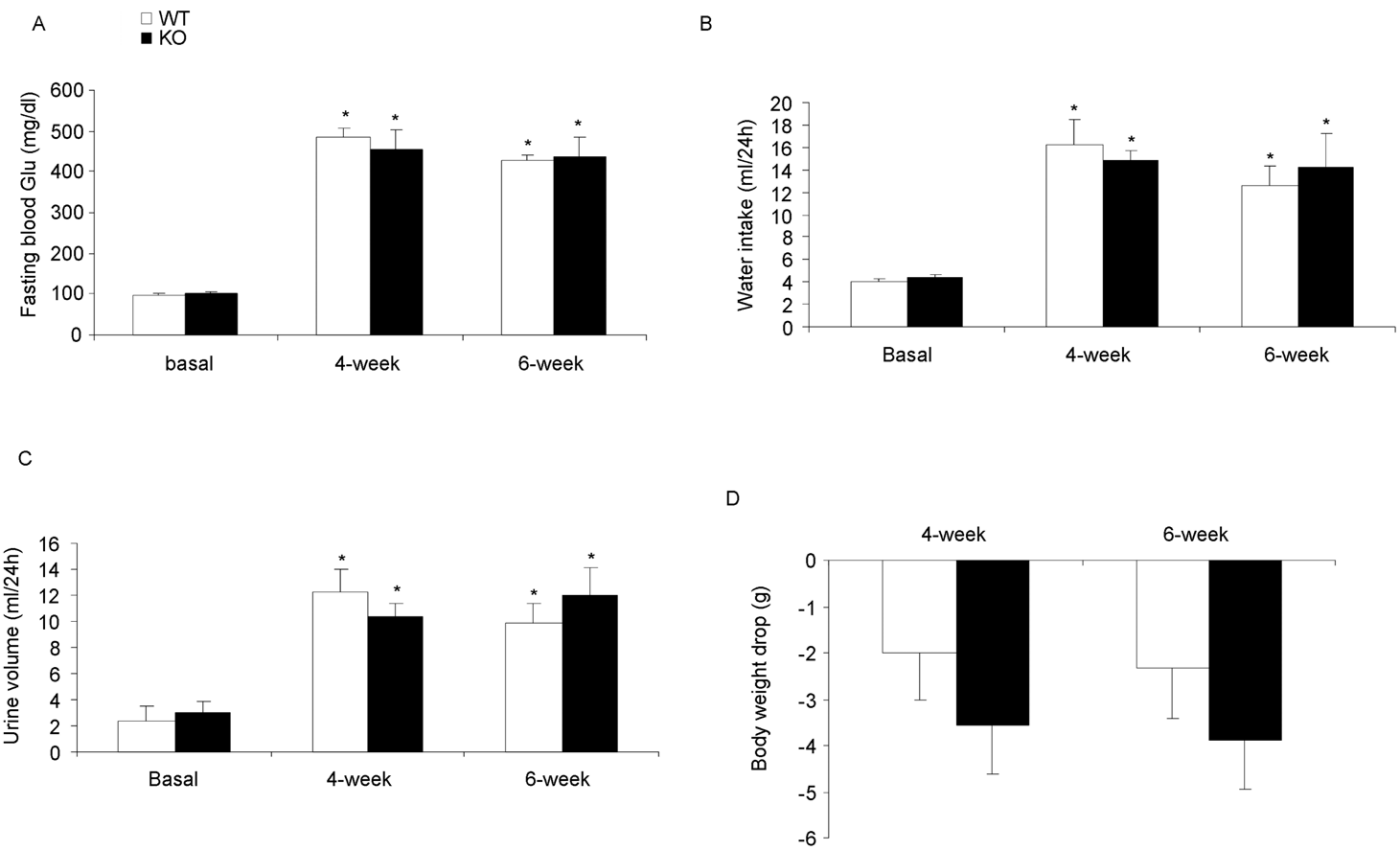
Evaluation of the onset of diabetes in mPGES-1 WT and KO mice. (A) Fasting blood glucose. (B) Water intake. (C) Urine volume. (D) Body weight changes. N = 6–9 in each group. *p<0.05 vs. basal. Data are mean ± SE.

We measured the fasting blood glucose (six hours fasting), urine volume, water intake and body weight at fourth and sixth week after STZ treatment. There was a significant increase of blood glucose in both WT and KO mice at fourth and sixth week after STZ injection (STZ vs. Control, p<0.01 in both WT and KO mice) without difference between two genotypes (4-week: WT/STZ 483.8±25.1 vs. KO/STZ 454.1±48.5 mg/dl, *p*>0.05; 6-week: WT/STZ 430.0±10.25 vs. KO/STZ 436.0±47.8 mg/dl, *p*>0.05) (Fig. 1A). Water intake and urine volume followed the similar pattern (Fig. 1B and C). After six weeks, a similar reduction of body weight about 2–3 g was observed in both genotypes. These data suggest that mPGES-1 did not affect the onset of STZ-induced diabetes.

### Evaluation of the diabetic kidney injury in mPGES-1 WT and KO mice

**Figure 2:**
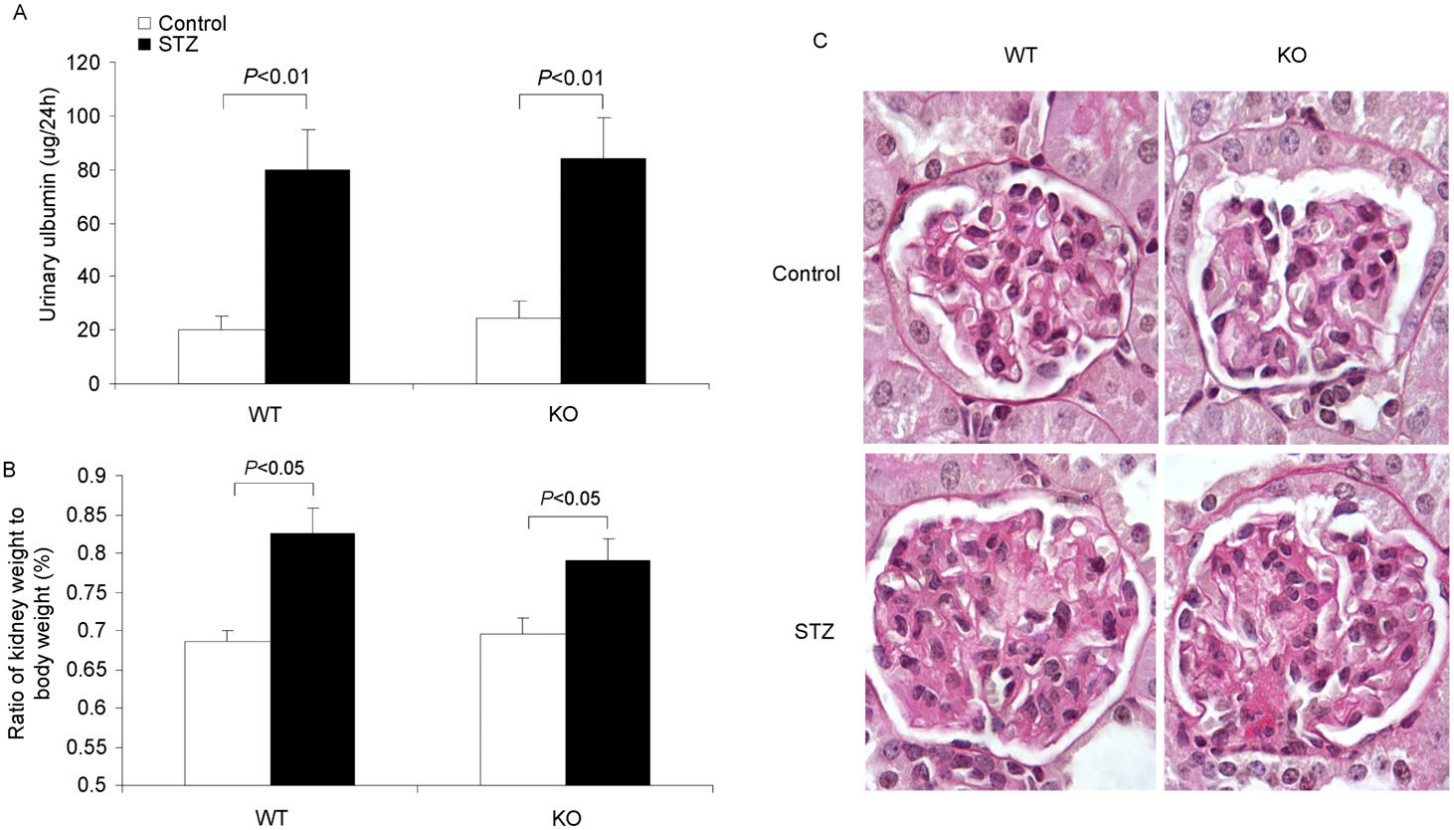
Evaluation of the diabetic kidney injury in mPGES-1 WT and KO mice. (A) The urinary albumin excretion after 6 weeks of diabetes. (B) Ratio of the kidney weight to the body weight (%). (C) PAS staining of the glomeruli after 6 weeks of diabetes. N = 6–9 in each group. Data are mean ± SE.

COX-2 and EP1/EP4 receptors have a detrimental role in diabetic nephropathy. Here we evaluated the effect of mPGES-1 deletion on diabetes-induced kidney injury via examining urinary albumin, the kidney weight and glomerular morphology. Both mPGES-1 WT and KO mice developed the comparable proteinuria after six weeks of STZ treatment (WT/Cont 20.3±4.7 vs. WT/STZ 79.61±15.1 ug/24 h, p<0.01; KO/Cont 24.15±6.9 vs. KO/STZ 84.12±15.1 ug/24 h, p<0.01; WT/STZ vs. KO STZ, p>0.05) (Fig. 2A). The kidney weight to the body weight ratio was also similar between WT and KO mice (WT/STZ 0.825±0.032 vs. KO/STZ 0.79±0.029% BW, *p*>0.05) (Fig. 2B). As for the glomerular morphology, the PAS staining showed the remarkable enlargement of the glomeruli and the increased mesangial area after 6 weeks of diabetes (Fig. 2C) with no difference between two genotypes (Fig. 2C). These results indicated that mPGES-1 deletion did not prevent the diabetic glomerular injury.

### Urinary PGE2 Excretion and Renal PGE2 content in diabetic mice

**Figure 3:**
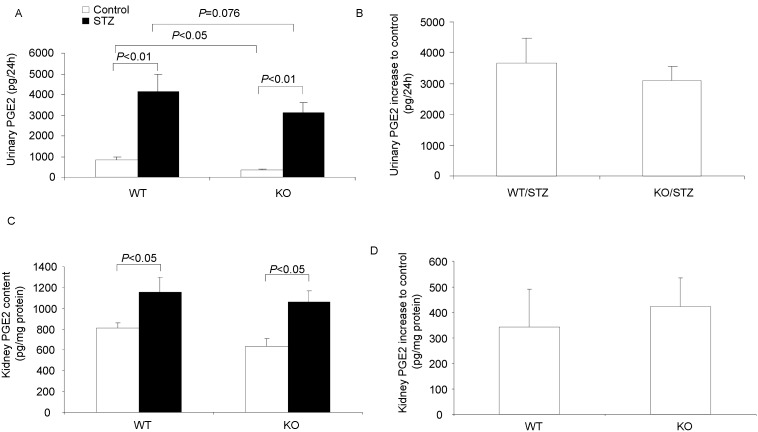
Urinary PGE2 excretion and renal PGE2 content in diabetic mice. (A) 24-hour urinary PGE2 excretion after 6 weeks of diabetes. (B) Increase of 24-hour urinary PGE2 excretion after 6 weeks of diabetes. (C) Kidney PGE2 content after 6 weeks of diabetes. (D) Increase of kidney PGE2 content after 6 weeks of diabetes. N = 6–9 in each group. Data are mean ± SE.

To further elicit the reason that mPGES-1 deletion did not affect the kidney injury in diabetic mice, we measured the urinary PGE2 excretion and kidney PGE2 content. By EIA assay, we found that mPGES-1 deletion had no effect on the diabetes-induced renal PGE2 production except for a lower baseline (urine: WT/STZ 3662.0±801.7 vs. KO/STZ 3086.5±465.4 pg/24 h, p>0.05; kidney: WT/STZ 857.3±369.0 vs. KO/STZ 1049.3±277.0 pg/mg protein, p>0.05) (Fig. 3A–D). These results indicated that mPGES-1 is not responsible for the renal PGE2 induction in the present model, which is very unexpected in consideration of previous evidences from our and other groups showing the critical role of mPGES-1 in mediating the PGE2 stimulation in various models [18]–[21], [23], [24], [28].

### Regulations of renal PGESs and 15-PGDH in diabetic mice

**Figure 4:**
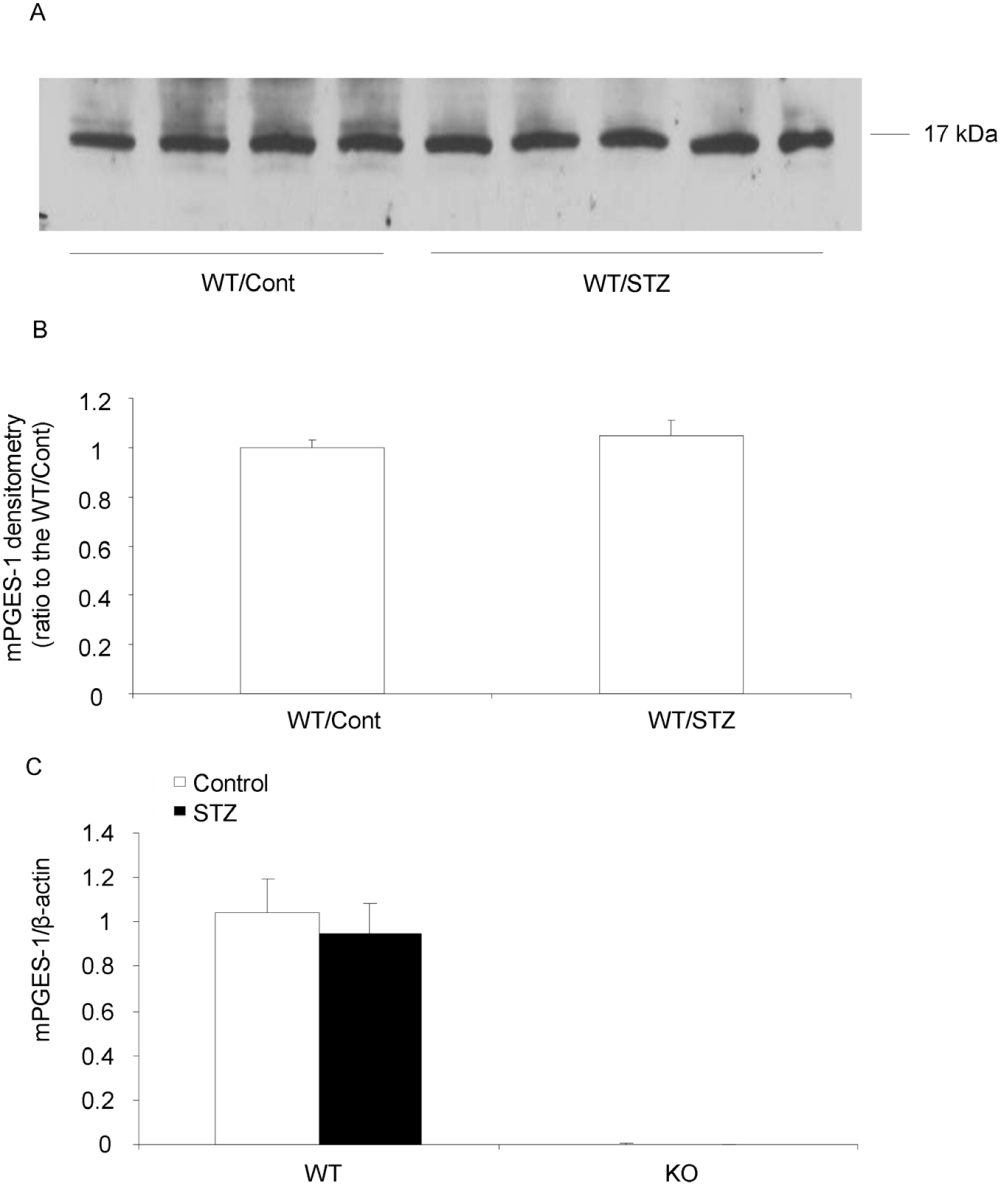
Expressions of mPGES-1 protein and mRNA in diabetic mice. (A) mPGES-1 protein analysis by Western blotting (n = 4–5 in each group). (B) Densitometry analysis of mPGES-1 Western blotting. (C) mPGES-1 mRNA expression by qRT-PCR (N = 6–9 per group). Data are mean ± SE.

**Figure 5:**
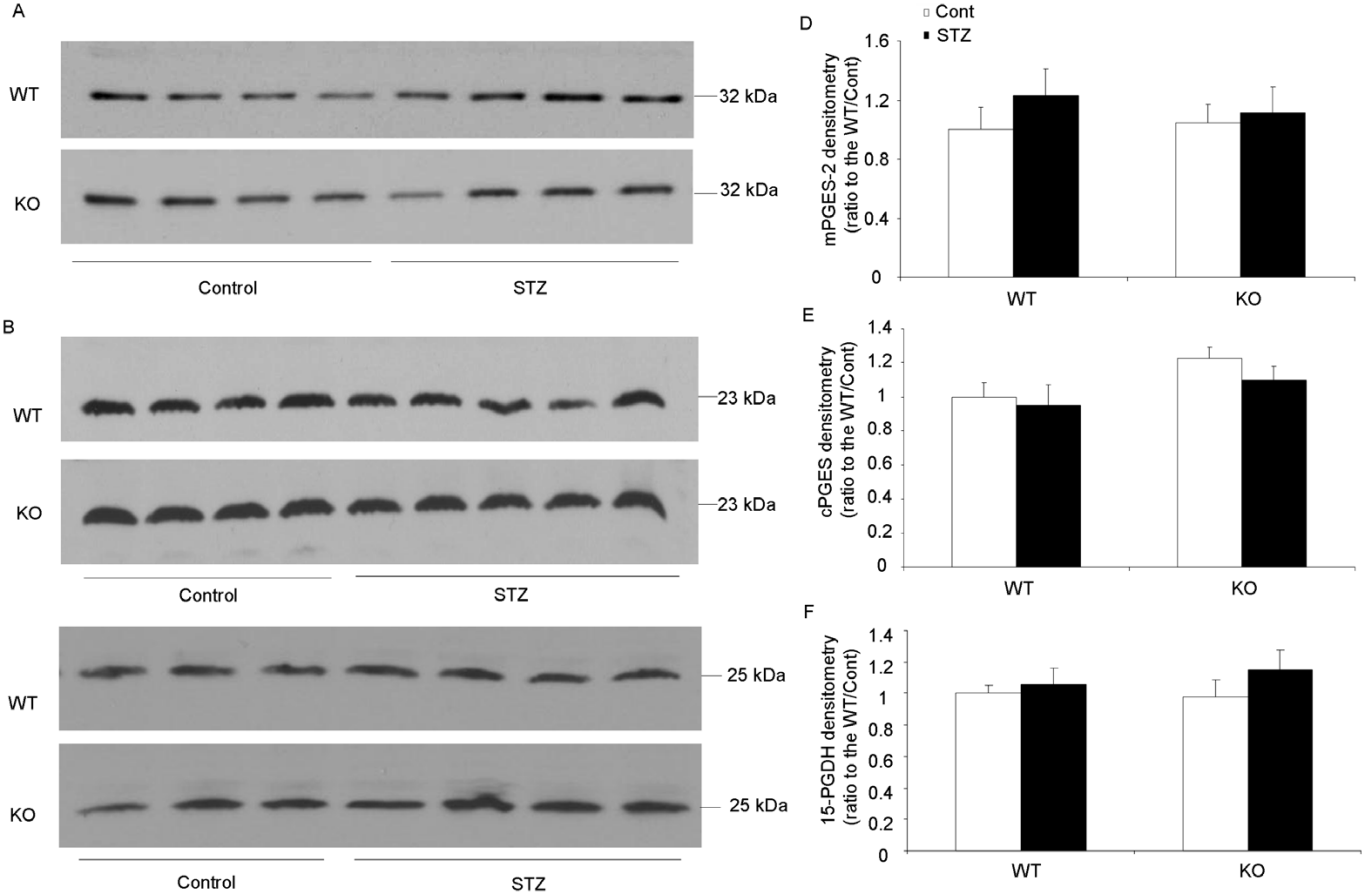
Protein expressions of mPGES-2, cPGES and 15-PGDH in diabetic mice. (A) mPGES-2 protein analysis by Western blotting (N = 4 per group). (B) cPGES protein analysis by Western blotting (N = 4–5 per group). (C) 15-PGDH protein analysis by Western blotting (N = 3–4 per group). (D) Densitometry analysis of mPGES-2 Western blotting. (E) Densitometry analysis of cPGES Western blotting. (F) Densitometry analysis of 15-PGDH Western blotting. Data are mean ± SE.

**Figure 6:**
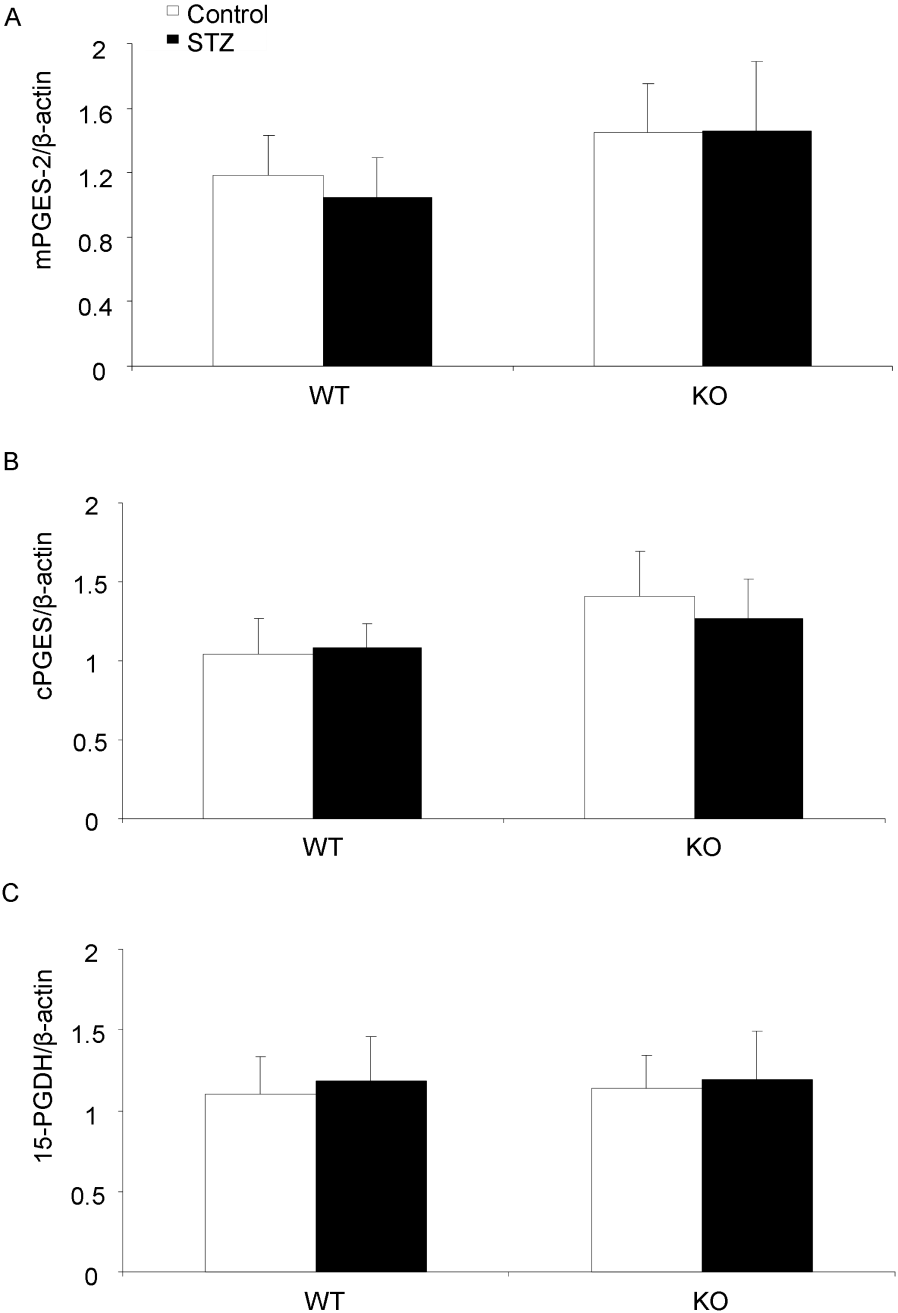
mRNA expressions of mPGES-2, cPGES and 15-PGDH in diabetic mice. (A) mPGES-2 mRNA expression by qRT-PCR (N = 6–9 per group). (B) cPGES mRNA expression by qRT-PCR (N = 6–9 per group). (C) 15-PGDH mRNA expression by qRT-PCR (N = 6–9 per group). Data are mean ± SE.

We further determined the mPGES-1 protein and mRNA levels in the kidney of control and diabetic mice. Consistent with the renal PGE2 level, renal mPGES-1 expression was not changed in this STZ diabetic mouse model (Fig. 4A–C). This stimulated our interest to test expression of other PGE2 synthesis enzymes. But neither mPGES-2 nor cPGES expression at protein and mRNA levels was altered in this model (Fig. 5A and B & Fig. 6A and B). The expression of 15-PGDH, an important enzyme responsible for degradation of PGs also remained unchanged at both protein and mRNA levels (Fig. 5C & Fig. 6C). These results excluded the role of mPGES-1 in mediating type-1 diabetes-induced renal PGE2 production and also did not favor the involvement of mPGES-2, cPGES and 15-PGDH in this process.

### Effects of celebrex on the proteinuria and kidney PGE2 induction

**Figure 7:**
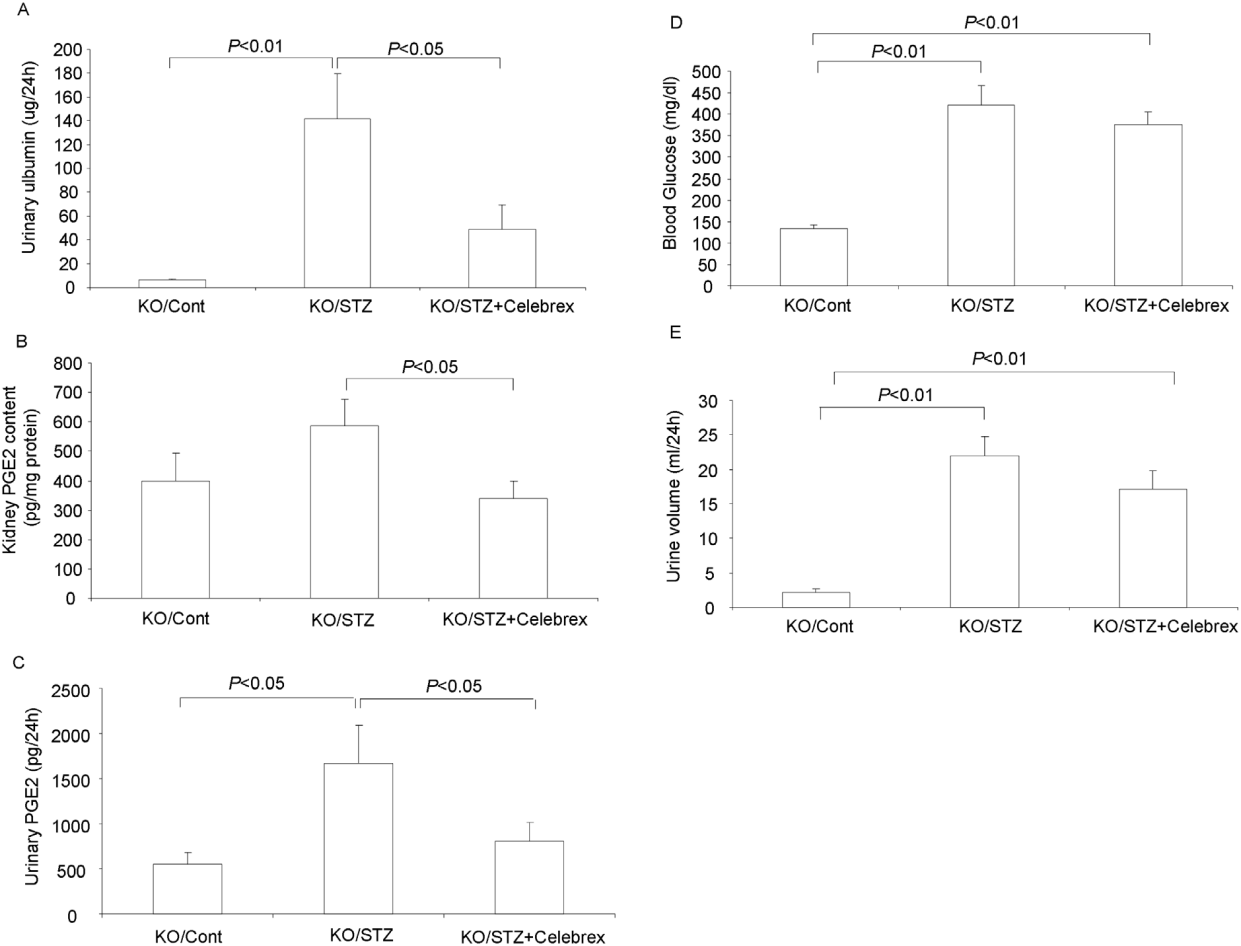
Effects of celebrex treatment on proteinuria and kidney PGE2 production in diabetic mPGES-1 KO mice. (A) Urinary ulbumin excretion (N = 5–6 per group). (B) Kidney PGE2 content (N = 5–6 per group). (C) Urinary PGE2 excretion. (D) Blood glucose levels (N = 5–6 per group). (E) Urine volume. Data are mean ± SE.

mPGES-1 deletion did not affect the renal PGE2 production or urinary albumin excretion in STZ diabetic mouse. To elucidate whether COX-2 plays a role in the renal PGE2 production and the proteinuria in these mPGES-1 KO mice with type-1 diabetes, we treated the diabetic KO mice with a selective COX-2 inhibitor celebrex. STZ treatment remarkably increased urinary albumin excretion in these KO mice, which was significantly attenuated by celebrex (KO/STZ 141.5±38.4 vs. KO/STZ + Celebrex 48.7±20.8 ug/24 h, p<0.05) (Fig. 7A). Meanwhile, Celebrex significantly suppressed the PGE2 production in diabetic mPGES-1 KO mice (Kidney: KO/STZ 588.7±89.2 vs. KO/STZ + Celebrex 340.8±58.7 ug/mg protein, *p*<0.05; Urine: KO/STZ 1667.6±421.4 vs. KO/STZ + Celebrex 813.6±199.9 pg/24 h, *p*<0.05) (Fig. 7B and C). Although celebrex showed its significant effect on both proteinuria and renal PGE2 production, it did not affect blood glucose (KO/STZ 421.4±44.4 vs. KO/STZ + Celebrex 375.8±28.8 mg/dl, *p*>0.05) (Fig. 7D) and urine volume (Fig. 7E) after six-week STZ treatment.

## Discussion

Although COX-2 inhibitors are widely used as the anti-inflammatory drugs in clinic, the numerous side effects, particularly the cardiovascular concerns, significantly limited its long-term use in patients. This situation instigates us to pursue the next generation of anti-inflammatory drugs. In light of its critical role in pain and inflammatory response, mPGES-1 is viewed as the most promising target for developing new anti-inflammatory drugs [28], [30]. To date, many mPGES-1 inhibitors have been generated and some of them are being under the clinical trials [31]–[33]. Therefore, it is very important to assess the role of mPGES-1 in different pathological processes.

Previously, numerous investigations revealed that COX-2 activity and PGE2 production are increased in the kidneys of diabetic animals [4],[7],[8], and the inhibition of COX-2 or antagonism of PGE2 receptor EP1 [2]–[10], [34] remarkably ameliorated the renal injury in STZ-induced diabetic animal models or diabetic patients with DN [35]. The present study was designed to assess the potential involvement of mPGES-1-derived PGE2 in the development of DN. mPGES-1 is very abundant in the kidney, with a significant higher level in the renal medulla than in the renal cortex, similar to COX-1 and COX-2. mPGES-1 expression can be detected in macula densa, distal convoluted tubule, collecting duct, and renal medullary interstitial cells [36]. A series of studies from our and other groups demonstrated a remarkable role of mPGES-1 deletion on PGE2 production in various models including lithium-induced NDI, water or salt loading, Ang II or DOCA-salt- hypertension, aldosterone escape, LPS or cisplatin-induced renal failure [16]–[24]. Therefore, it is worthwhile to evaluate that whether mPGES-1 serves as a valuable target for DN treatment.

In present study, mPGES-1 deletion did not affect the blood glucose level in these diabetic mice, which demonstrated a paralleled diabetes onset and suggested a similar pancreatic β-cell injury in mPGES-1 WT and KO mice. This result agrees with a recent study showing that mPGES-1 in islet played no role in IL-1β-caused inhibitory effect on insulin secretion [37]. In agreement with this observation, mPGES-1 deletion did not suppress renal PGE2 induction in STZ-induced diabetic mice. Accordingly, the expression of mPGES-1 in the kidney remained unchanged in WT diabetic mice compared with the WT controls. In line with these negative phenotypes, the mPGES-1 deletion did not alter the renal phenotypes in response to the STZ-induced type-1 diabetes as shown by the comparable proteinuria, kidney hypertrophy andglomerulopathy. These results suggested no role mPGES-1 may play in diabetes-associated kidney injury and PGE2 production, at least in STZ diabetic mouse.

To further elicit the possible sources of renal PGE2 production in present type-1 diabetic model, we measured renal protein levels of mPGES-2, cPGES and 15-PGDH. The mPGES-2 is synthesized as a Golgi membrane-associated protein, and spontaneous cleavage of the N-terminal hydrophobic domain leads to the formation of mature cytosolic enzyme [38]. No specific phenotype was found in the mPGES-2 KO mice, as well as the basal PGE2 levels in different organs [25]. LPS stimulated PGE2 release from mPGES-2 KO macrophages was also unaltered compared with WT cells [25]. As for cPGES, it is abundant in the cytosol of various tissues and cells [39]. The expression of cPGES is not changed by proinflammatory stimuli in most cases with a few exceptions [39], [40]. Similar as mPGES-2 KO mice, cPGES deletion can not lower the PGE2 levels in vivo. Therefore, although mPGES-2 and cPGES presented their in vitro property of PGE2 generation, their in vivo role of PGE2 production is still uncertain. In present study, both mPGES-2 and cPGES protein levels were not affected in kidneys of STZ diabetic mouse. This suggested that both mPGES-2 and cPGES were unlikely involved in the PGE2 production in this model.

To further test whether PGE2 degradation contributes to the renal PGE2 level in these diabetic mice, we examined the 15-PGDH protein expression by Western blotting. 15-PGDH is a member of short-chain dehydrogenase/reductase (SDR) family catalyzing the PGE2 catabolic pathway [41]. Its functional role in cardiovascular and pulmonary systems has been extensively studied. However, its pathophysiological role in kidney remains poorly understood. In present study, we did not find any alteration of renal 15-PGDH protein level following 6-week diabetes in both WT and KO mice. But this result can not entirely rule out the involvement of 15-PGDH in renal PGE2 generation without the evidence of enzyme activity change.

Finally, we treated diabetic mPGES-1 KO mice with a selective COX-2 inhibitor celebrex and found that inhibition of COX-2 significantly reduced the kidney PGE2 content, as well as the urinary albumin level, without affecting the hyperglycemia. These results demonstrated that COX-2 contributed to the diabetes-related kidney PGE2 induction and was significantly involved in the diabetic proteinuria.

In summary, the present study examined the role of mPGES-1 along with COX-2 in diabetic kidney injury and PGE2 overproduction. Although mPGES-1-derived PGE2 played an important role in many pathophysiological conditions, we found no evidence supporting involvement of this enzyme in diabetes-related PGE2 production and glomerular injury, or the onset of STZ-induced diabetes, at least in the current mouse model. This discrepancy of mPGES-1 regulation and function between the type-1 diabetes-associated kidney injury and other kidney diseases may be due to the different pathogenic mechanisms among various diseases. Moreover, renal expression of other components of the PGE2 synthesis and degradation pathway including mPGES-2, cPGES, and 15-PGDH remained unaltered in the diabetic kidney. These negative observations contrast sharply with the remarkable effect of COX-2 inhibition on kidney injury and PGE2 levels. Together, these results suggest that an as yet unidentified PGE2 synthase may exist to couple with COX-2 to mediate renal PGE2 production in diabetes.
